# Langfristige Abheilung eines therapierefraktären Ulcus cruris venosum nach kruraler Fasziektomie und Spalthauttransplantation

**DOI:** 10.1007/s00105-020-04659-3

**Published:** 2020-07-27

**Authors:** Monika Kleinhans, Ingo Stoffels, Joachim Dissemond

**Affiliations:** Klinik und Poliklinik für Dermatologie, Venerologie und Allergologie, Universitätsklinikum Essen, Hufelandstr. 55, 45122 Essen, Deutschland

**Keywords:** Chronische Wunde, Shave-Therapie, Krurale Fasziektomie, Wundgrundkonditionierung, Mesh-graft-Technik, Chronic wounds, Shave therapy, Crural fasciectomy, Wound bed preparation, Mesh graft technique

## Abstract

Bei einer 59-jährigen adipösen Patientin bestand seit 1986 ein schmerzhaftes, chronisches Ulcus cruris. Wir stellten die Diagnose eines Ulcus cruris venosum bei funktioneller chronischer venöser Insuffizienz im Rahmen der Adipositas per magna (Dependency-Syndrom) und arthrogenem Stauungssyndrom bei Immobilität. Obwohl in der Vergangenheit bereits eine Krossektomie und Stripping der V. saphena magna und mehrfach eine Shave-Therapie mit Spalthauttransplantation nach entsprechender Wundgrundkonditionierung erfolgten, persistierte die Wunde. Bei therapierefraktärem Verlauf und hohem Leidensdruck mit ausgeprägten Schmerzen war es 2012 der Wunsch der Patientin, das Bein zu amputieren. Als Alternative zu der Amputation führten wir eine krurale Fasziektomie durch und nach erfolgter Wundgrundkonditionierung mittels 2 Zyklen einer Vakuumtherapie eine anschließende Versorgung des Defektes mit einer Spalthaut in Mesh-graft-Technik. Die Operation verlief ohne Komplikationen, und die Haut heilte vollständig ein. Bei der aktuellen Wiedervorstellung 7 Jahre nach dem Eingriff zeigte sich trotz weiterhin bestehender Adipositas per magna und Immobilität eine vollständige Heilung des zuvor therapierefraktären Ulcus cruris venosum. Dieser Fallbericht zeigt eindrucksvoll, dass insbesondere bei ansonsten therapierefraktären Verläufen des Ulcus cruris venosum eine krurale Fasziektomie erwogen werden kann.

## Abstract (en)

A 59-year-old obese woman had been suffering from a painful, chronic leg ulcer since 1986. We diagnosed a venous leg ulcer due to functional chronic venous insufficiency in the context of obesity per magna (dependency syndrome) and arthrogenic congestion syndrome due to immobility. Although a high ligation and stripping of the great saphenous vein and several so-called shave therapies with split skin transplantation after appropriate wound bed preparation had been previously performed, the wound persisted. In 2012, the patient wanted to amputate her leg because the course of wound healing was so refractory to therapy and she suffered from severe pain. As an alternative to amputation, we performed a crural fascietomy and, after a wound bed preparation with two cycles of negative pressure wound therapy, we covered the defect with a split skin graft using the mesh graft technique. The surgery could be performed without complications and the skin healed completely. At the current presentation 7 years after the intervention, complete healing of the previously therapy-refractory venous leg ulcer was shown despite the persistent obesity per magna and immobility. This case report shows that a crural fasciectomy should be considered, especially in the case of otherwise therapy-refractory courses of venous leg ulcers.

## Anamnese

Es stellte sich im Oktober 2019 eine 59-jährige Patientin mit einem ambulant therapierefraktären, schmerzhaften Ulcus cruris des linken Unterschenkels sowie einem juckenden Ekzem des rechten Unterschenkels zur stationären Aufnahme vor. Die adipöse Patientin ist u. a. wegen einer Deformität der Füße und Arthrose weitestgehend immobil und sitzt die größten Teile des Tages ohne Kompressionstherapie in einem Rollstuhl.

Seit 1986 bestand ein schmerzhaftes Ulcus cruris rechts; seit 2005 traten auch wiederholt Ulzerationen am linken Unterschenkel auf. Therapeutisch erfolgten aufgrund des Ulcus cruris venosum u. a. eine Krossektomie der V. saphena magna rechts (extern) sowie chirurgische Wunddébridements, Shave- und Vakuumtherapien sowie mehrfach Hauttransplantationen ohne längerfristigen Erfolg. Die stationär durchgeführten Kompressionstherapien mittels Mehrkomponentenkompressionssystemen wurden bei unzureichender Adhärenz ambulant nicht konsequent fortgeführt. Begleitende Wundinfekte wurden mehrfach systemisch antibiotisch therapiert.

## Klinischer Befund

Bei der aktuellen Vorstellung zeigte sich am linken Malleolus medialis ein ca. 2 × 1 cm großes Ulkus ohne Anhalt für einen Wundinfekt. Am rechten Unterschenkel bestanden eine teils erosive Stauungsdermatitis sowie vereinzelte hämorrhagische Krusten. Es zeigte sich eine ausgeprägte Fehlstellung der Füße beidseits mit Valgusstellung, Digitus pedis quintus superductus sowie Digitus pedis secundus superductus. An beiden Beinen imponierte eine ausgeprägte Purpura jaune d’ocre sowie Atrophie blanche. Zudem bestanden am linken Unterschenkel eine Dermatoliposklerose und Stauungsdermatitis als weitere klinische Zeichen einer chronischen venösen Insuffizienz (CVI). Auf der visuellen Analogskala (VAS) bestanden Schmerzen in einer Stärke von 3/10.

**Figure 1:**
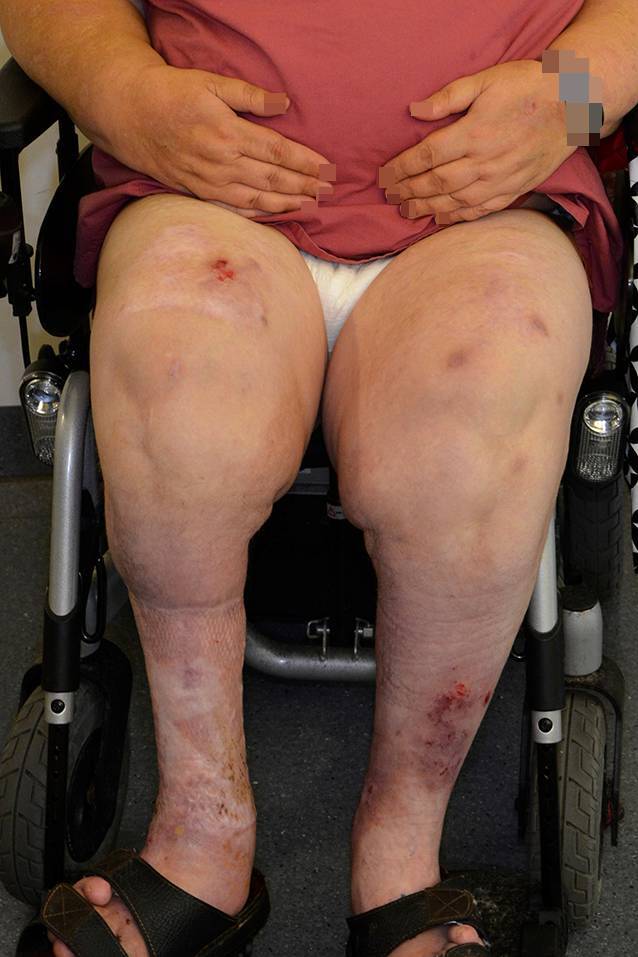

Bei einer Größe von 170 cm und einem Gewicht von 139 kg mit einem BMI (Body-Mass-Index) von 48,1 kg/m^2^ bestand eine Adipositas per magna (Abb. 1). Medikamentös behandelt wurden ein Diabetes mellitus Typ 2, eine Hypertriglyzeridämie und eine arterielle Hypertonie.

## Diagnose

Bei der von uns beschriebenen Patientin zeigte sich weder 2012 noch 2019 duplexsonographisch ein ausgeprägter Venenschaden. Eine periphere arterielle Verschlusskrankheit (pAVK) wurde wiederholt durch apparative Messungen der arteriellen Verschlussdrücke ausgeschlossen. Mehrfach wurden Biopsien entnommen, um beispielsweise eine zugrunde liegende Neoplasie auszuschließen.

Da klinisch die typischen Zeichen einer CVI bestanden, stellten wir die Diagnose eines Ulcus cruris venosum bei funktioneller CVI (Stadium C_6_ E_s_ A_D13–16_ P_n_ entsprechend der CEAP-Klassifikation) im Rahmen der Adipositas per magna (Dependency-Syndrom) und arthrogenem Stauungssyndrom bei Immobilität.

## Therapie und Verlauf

**Figure 2:**
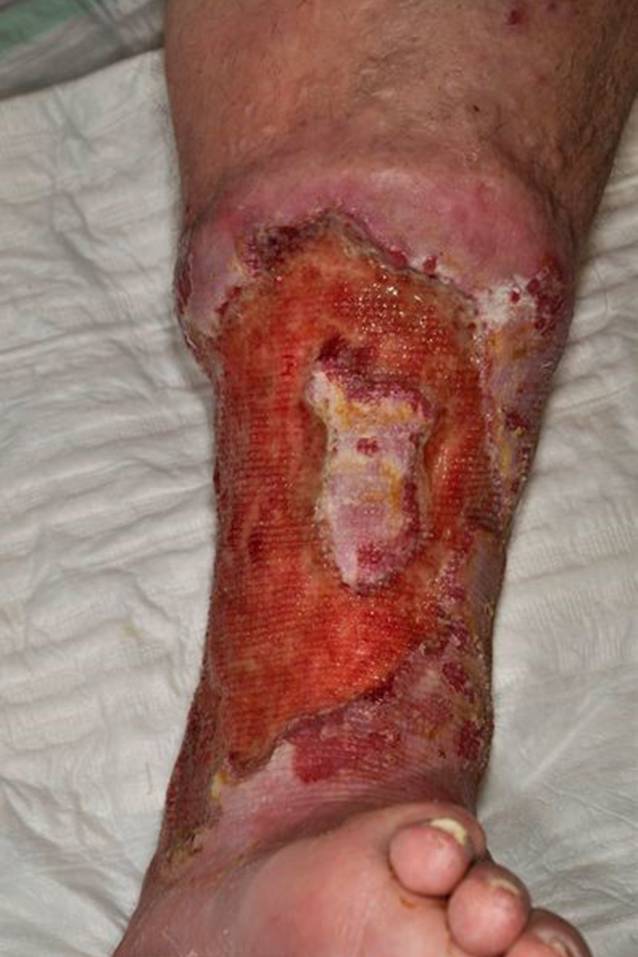

**Figure 3:**
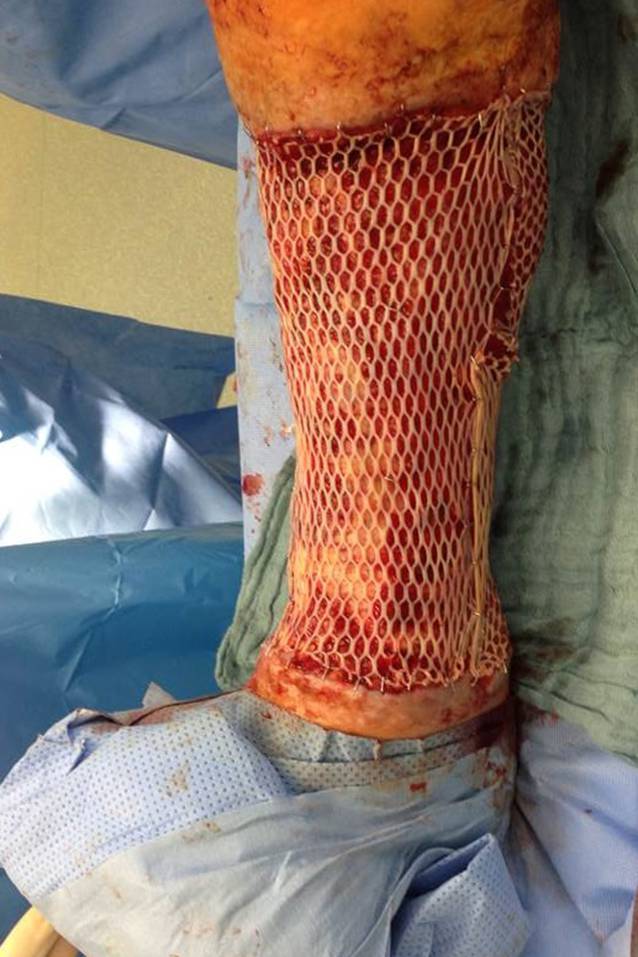

Bei sehr hohem Leidensdruck aufgrund des therapierefraktären Verlaufs und ausgeprägten Schmerzen (VAS 7/10) äußerte die Patientin 2012 den Wunsch nach Amputation des rechten Unterschenkels (Abb. 2). Nach Aufklärung über die zur Verfügung stehenden Behandlungsalternativen wurde bei der Patientin eine krurale Fasziektomie mit vollständiger Resektion des nekrotischen Gewebes einschließlich der nekrotischen Sehnen und der Faszien durchgeführt. Anschließend fand nach Wundgrundkonditionierung mit 2 Zyklen einer Vakuumtherapie die chirurgische Defektversorgung mittels Spalthaut in Mesh-graft-Technik statt (Abb. 3). Bereits 4 Wochen postoperativ zeigte sich eine gute Einheilung des Transplantates. In ambulanten Wiedervorstellungen imponierte eine vollständige Abheilung des zuvor bestehenden therapierefraktären Ulkus am rechten Unterschenkel.

## Diskussion

Eine der häufigsten Manifestationen chronischer Wunden in Europa ist das Ulcus cruris. Die hierfür zugrunde liegenden Ursachen können sehr vielfältig sein. Seltenere Differenzialdiagnosen sind beispielsweise Vaskulitis, Pyoderma gangraenosum, Kalziphylaxie oder Infektionskrankheiten. Mit etwa 80 % ist die CVI in Deutschland die häufigste Ursache für ein chronisches Ulcus cruris [7]. Der erstmalig 1957 von van der Molen beschriebene Begriff der CVI fasst dabei alle klinischen Veränderungen der Haut und Unterhaut im Rahmen einer chronischen Venenerkrankung zusammen. Meist resultiert eine CVI aus einem postthrombotischen Syndrom (PTS), einer Varikose oder einer Gefäßmalformation. Man geht davon aus, dass ca. 1–2 % der Patienten mit einer CVI im Laufe ihres Lebens ein Ulcus cruris venosum entwickeln. Weitere zunehmend diskutierte Ursachen sind funktionelle Störungen des Venensystems, wie beispielsweise das Adipositas-assoziierte Dependency-Syndrom (Tab. 1). Anders als bei der klassischen CVI bestehen hier keine Klappeninsuffizienzen oder Obstruktionen. Bei adipösen Patienten können ähnliche strukturelle Veränderungen der Venenwände und Venenklappen vorliegen, ohne dass der typische venöse Reflux besteht [1]. Einerseits spielt hier die Immobilität und dadurch fehlende Muskel-Venen-Pumpe eine Rolle, andererseits führen das viele Sitzen und das abdominelle Fett zu einem Druckanstieg in den Beinvenen [2]. Durch das sog. arthrogene Stauungssyndrom (Tab. 1) entsteht zudem eine Versteifung der Sprunggelenke, wodurch die Muskel-Venen-Pumpe nicht genügend aktiviert wird. Es kommt zu einem funktionellen Ausfall der peripheren Venenpumpen [5]. Ein weiteres bei der hier beschriebenen Patientin diskutiertes Problem war das sog. chronische venöse Kompartmentsyndrom (Tab. 1). Hiermit wird ein Krankheitsbild beschrieben, bei dem es zu einem Druckanstieg im Kompartiment durch die Dermatolipofasziosklerose kommt. Durch diese Sklerosierung kann sich die Faszie den Muskelkontraktionen nicht mehr anpassen, es resultiert eine Minderdurchblutung [4]. Es kann dann zu Ulzerationen kommen, die sich oft zirkulär um den Unterschenkel als sog. Manschetten- oder Gamaschenulkus entwickeln.

**Table 1:** 

Terminus	Definition
Dependency-Syndrom	Adipositas-assoziierte chronische Veneninsuffizienz. Die venöse Hypertonie wird insbesondere durch Kompression der Beinvenen durch die abdominelle Fettschürze verursacht
Arthrogenes Stauungssyndrom	Venöse Stauung bei Inaktivität der Muskel-Venen-Pumpe durch Versteifung der Sprunggelenke
Chronisches venöses Kompartmentsyndrom	Erhöhung des intrakompartimentären Drucks durch Sklerose der Fascia cruris
Shave-Therapie	Schichtweise tangentiale Resektion des epifaszialen nekrotischen und sklerotischen Ulkusgewebes
Krurale Fasziektomie	Resektion der Fascia cruris unter Einbeziehung des gesamten nekrotischen und sklerotischen Gewebes

Goldstandard der Diagnostik bei Verdacht auf CVI ist die Farbduplexsonographie des Beinvenensystems bei stehenden Patienten. Bei Patienten mit funktioneller CVI kann dieser Untersuchungsbefund normal sein, während das abdominelle Fett im Sitzen eine Kompression der Beinvenen verursacht [2, 8]. Eine weitere diagnostische Option ist die blutige Venendruckmessung. Hier wird eine oberflächliche Fußrückenvene punktiert und der Venendruck in Ruhe und bei Aktivierung der Muskelpumpe ermittelt. Beim Gehen sollte es zu einem Abfall des Drucks kommen, ist dies nicht der Fall, handelt es sich um eine ambulatorische venöse Hypertonie.

Therapeutisch ist bei den meisten Patienten mit Ulcus cruris venosum ein Débridement essenziell, bei dem avitales Gewebe entfernt wird. Anschließend werden eine moderne, an den Phasen der Wundheilung angepasste Wundbehandlung sowie eine Kompressionstherapie durchgeführt. Es sollte immer auch abgeklärt werden, ob eine interventionelle Therapie am Venensystem sinnvoll ist. Trotz adäquat durchgeführter Behandlung kann es zu therapierefraktären Verläufen kommen. Insbesondere bei sklerotischem Wundgrund ist die lokale Ulkuschirurgie in Form einer sog. Shave-Therapie (Tab. 1) die Methode der ersten Wahl. Erstmalig beschrieben wurde dieses Verfahren 1996 durch Prof. Wilfried Schmeller [9]. Im Vergleich zu einem konventionellen Wunddébridement, kommt es hier zu der großflächigeren und tieferen Abtragung von sklerotischem Gewebe in und um die Wunde. Dieses sklerotische Gewebe wird so tief abgetragen, bis der Wundgrund aus vitalem Gewebe besteht. Diese Abtragung kann bis zu den Muskelfaszien notwendig sein.

Wenn zudem ein chronisches venöses Kompartmentsyndrom vorliegt, kann auch die Durchführung eines chirurgischen Eingriffs an der Faszie erwogen werden. Als Erstbeschreiber der paratibialen Fasziotomie gilt Prof. Wolfgang Hach [6]. Bei dieser Methode kommt es zu der Eröffnung eines Kompartimentes durch eine Spaltung der Fascia cruris. So kann die Muskulatur von pathologischen Drücken entlastet werden, und es kommt zu einer Erholung der muskulären Gewebestrukturen. Bei der kruralen Fasziektomie hingegen wird die Faszie vollständig unter Einbeziehung des gesamten nekrotischen und sklerotischen Gewebes reseziert. Die krurale Fasziektomie ist wesentlich traumatischer und aufwendiger als beispielsweise eine Shave-Therapie; mögliche Risiken sind u. a. Gefäß- und Nervenverletzungen. Eine weitere Möglichkeit ist die durch Homans erstmalig 1916 durchgeführte partielle Fasziektomie, bei der das Ulkus lokal einschließlich der betroffenen Faszie exzidiert wird.

Die Effektivität der paratibialen Fasziotomie und der kruralen Fasziektomie konnte bislang nur in kleinen Kollektiven wissenschaftlich untersucht werden. Hierbei zeigten sich bei der paratibialen Fasziotomie Abheilungsraten von mehr als 90 % [3]. In einer vergleichenden Untersuchung von insgesamt 21 Patienten verzeichnete man mehrere Jahre nach Shave-Therapie eine Rezidivfreiheit von 70–80 %, während durch die krurale Fasziektomie eine Rezidivfreiheit von 50 % erreicht wurde [10]. Einschränkend ist anzumerken, dass in dieser telefonisch durchgeführten retrospektiven Untersuchung ein Bias enthalten sein kann, da die Indikationen für die jeweils durchgeführten Untersuchungen sehr unterschiedlich gewesen sein könnten und somit die Gruppe der Patienten mit kruraler Fasziektomie therapierefraktärer und schwerwiegender war.

**Figure 4:**
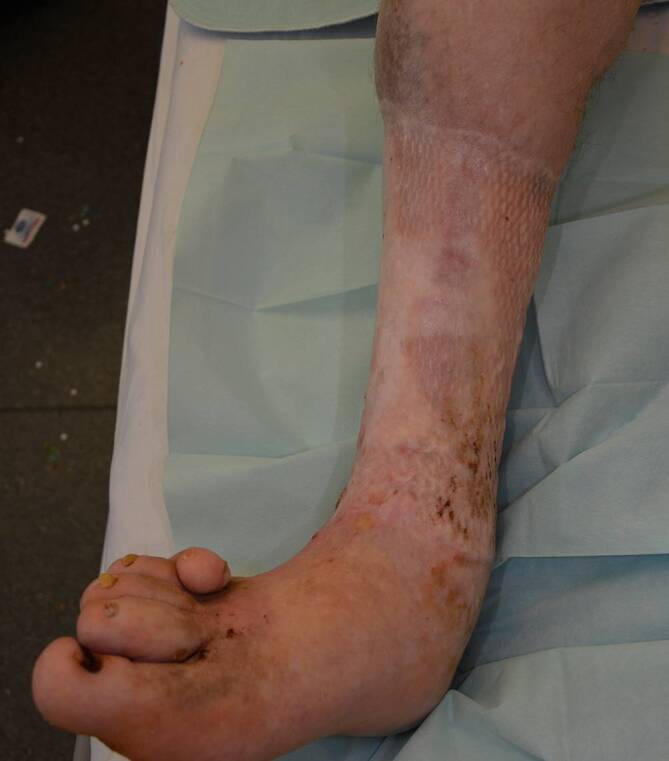

Zusammenfassend betrachtet gibt es bislang wenig Evidenz zu den langfristigen Erfolgen der kruralen Fasziektomie bei Patienten mit Ulcus cruris venosum. Unser Fallbericht zeigt einen sehr langen Leidensweg über Jahrzehnte, bei dem schlussendlich die krurale Fasziektomie mit nachfolgender Spalthautdeckung zu einer vollständigen Abheilung des zuvor therapierefraktären Ulcus cruris venosum geführt hat (Abb. 4). Dieser Erfolg hält nun seit 7 Jahren an, obwohl die Patientin weder an Gewicht abgenommen hat, weiterhin immobil war und keine Kompressionstherapie akzeptierte. Dieser Fallbericht soll daher die heute selten genutzte Therapieoption der krurale Fasziektomie bei therapierefraktären Verläufen eines Ulcus cruris venosum verdeutlichen.

## Fazit für die Praxis

- Bei adipösen und/oder immobilen Patienten kann eine funktionelle chronische venöse Insuffizienz (CVI) bis hin zu einem Ulcus cruris venosum auftreten.
- Bei therapierefraktären Verläufen eines Ulcus cruris venosum sollte nach Sanierung insuffizienter epifaszialer Venenabschnitte und Perforansinsuffizienzen die Behandlungsoption einer Shave-Therapie besprochen werden.
- Wenn nach diesen interventionellen Maßnahmen die Wunden weiterhin persistieren, kommt auch eine krurale Fasziektomie als Intervention in Betracht.
